# Different bacterial growth of major mastitis pathogens after coculturing with *Staphylococcus chromogenes* and *Staphylococcus hominis* in milk *in vitro*

**DOI:** 10.3389/fmicb.2025.1707938

**Published:** 2025-11-12

**Authors:** A. Chuasakhonwilai, P. Keeklangdon, W. Chaisri, D. Saipinta, K. Photiboon, D. Kaewmuangma, W. Anuphom, M. Intanon, W. Suriyasathaporn

**Affiliations:** 1 Office of Research Administration, Chiang Mai University, Chiang Mai, Thailand; 2 School of Veterinary Medicine, Faculty of Veterinary Medicine, Chiang Mai University, Chiang Mai, Thailand; 3 Research Center of Producing and Development of Products and Innovations for Animal Health and Production, Chiang Mai University, Chiang Mai, Thailand; 4 Cambodia Campus, Asian Satellite Campuses Institute, Nagoya University, Nagoya, Japan

**Keywords:** mastitis, coculture, non-aureus staphylococci, major mastitis pathogens, milk

## Abstract

Non-aureus staphylococci (NAS) mainly cause subclinical bovine mastitis and often either spontaneously resolve or become persistent and challenging to treat. Additionally, coculturing with certain NAS strains may provide a protective effect against more severe pathogens, becoming the idea of a vaccine for treatment. Testing coinfection interactions directly in milk, the natural nutrient environment, is more appropriate than using standard culture media. Understanding these interactions may offer new insights into infection dynamics. Therefore, this study aimed to determine the pattern of bacterial growth in milk for the major mastitis pathogens, including *S. uberis*, *S. agalactiae*, and *S. aureus*, in single culture or coculture with certain *S. chromogenes* and *S. hominis*, which have been proven as NAS protective strains *in vitro*. For major mastitis pathogens, three mastitis strains of each pathogen were included. The stock major bacteria were prepared for 10^5^ CFU/mL, while the stocked minor bacteria were adjusted to 10^9^ CFU/mL. Cultures were incubated at 37 °C. After incubation for 0, 8, 12, and 24 h, all samples were collected to determine bacterial growth using selective media. Logarithm bacterial counts were used for statistical analysis using generalized mixed linear models. Results demonstrated distinct growth dynamics of major and minor mastitis pathogens in milk. For the first 12 h of incubation, marked increases were observed for major pathogens, but minor pathogens continued to increase slightly. *S. aureus* had the highest growth rate. The growth rate of *S. uberis* was higher when cocultured with *S. chromogenes* than in its single culture but *S. agalactiae* was higher when cocultured with both minor pathogens. No significant difference was found for the growth rate of *S. aureus* after coculture. Except for the higher growth of *S. hominis* cocultured with *S. aureus*, the growth of *S. chromogenes* in both single and coculture with most major pathogens was significantly higher than that of *S. hominis*. In conclusion, the *in vitro* proven protective NAS strains could survive in milk after coculture with major pathogens. This method can be applied as a tool to evaluate the interaction between mastitis pathogens and weakened pathogens of live-attenuated vaccines for treating mastitis.

## 1 Introduction

Mastitis, an inflammation of the mammary gland, is one of the most common and economically significant diseases in dairy cattle worldwide. Epidemiological studies indicate that *S. aureus* is the most frequently isolated mastitis pathogen (25%), followed by coagulase-negative staphylococci (20%), *E. coli* (11%), *S. agalactiae* (9%), and *S. uberis* (9%) (Krishnamoorthy et al., 2021). It leads to reduced milk yield, impaired milk quality, increased treatment costs, and significant economic losses to the dairy industry. Bacterial pathogens associated with mastitis are diverse, with more than 20 species reported globally (Zhao et al., 2025). These pathogens are commonly categorized into major and minor groups according to the severity of infection they cause. Major pathogens, such as *S. aureus, S. agalactiae,* and *S. uberis*, can cause clinical mastitis with visible udder inflammation, abnormal milk appearance, and systemic symptoms. In contrast, minor pathogens, such as non-aureus staphylococci (NAS), including *S. chromogenes* and *S. hominis*, are often associated with subclinical mastitis and often either spontaneously resolve or become persistent and challenging to treat. Different species and strains of NAS differ in their epidemiology, pathogenicity, virulence, ecology, and host adaptation, and antimicrobial resistance profiles (for reviews, see De Buck et al., 2021, Kerro Dego and Vidlund, 2024). Additionally, coculturing certain NAS strains using a conventional method provided a protective effect against more severe pathogens, such as *S. aureus* and *S. agalactiae* (Srithanasuwan et al., 2023), suggesting a possible nonspecific pathogen vaccine for treatment. To alleviate the loss from mastitis, live-attenuated vaccines against specific mastitis pathogens in cows are generally available. Examples include a live-attenuated *S. aureus* small-colony variant (SCV) vaccine that enhances protection and immunity responses, as well as another live vaccine against mastitis caused by *S. uberis*. Live vaccines work by having the weakened pathogen replicate in the animal, triggering a robust immune response and building immunological memory to protect against infection. Testing those weakened pathogens in an udder environment, such as milk, would be ideal for developing those nonspecific and specific vaccines.

Inadequate control of mastitis promotes intramammary infections (IMI) involving multiple bacterial species, often leading to mixed infections, with NAS being the most commonly isolated organisms (Sophorn et al., 2025). Additionally, mixed infections were detected through PCR in non-viable organisms that persist long after inactivation. Mixed infection is characterized by the simultaneous presence of more than one bacterial species within the same quarter, highlighting ecological interactions such as competition, coexistence, or sequential colonization. While various species may initially colonize the mammary gland, competitive mechanisms frequently result in the dominance of a single pathogen, and recurrent or successive infections are often observed in herds with poor mastitis management. Recent coculture studies involving mastitis-associated bacteria further support this idea: for instance, *S. chromogenes* inhibited *S. agalactiae*, whereas certain strains of *S. hominis* and *S. simulans* demonstrated resistance to both *S. aureus and S. agalactiae* (Srithanasuwan et al., 2023). However, this study utilized standard culture media, where bacteria compete to survive on the surface of agar. In udder, milk is comprised of a high amount of nutrition, that are sufficient for bacterial growth. The results of this previous study might not completely explain intramammary infection in the milk environment.

Therefore, this study was conducted to determine the protective effect of the proven NAS protective strains against major pathogens *in vitro* in the milk environment. The patterns of bacterial growth in the milk environment for the major mastitis pathogens, including *S. uberis, S. agalactiae,* and *S. aureus*, were examined both in single culture and in coculture with the proven protective strains of *S. chromogenes* and *S. hominis*. The understanding of single and mixed intramammary infections in this study could inform the bacterial growth pattern in milk, which might differ from that on standard culture media. It could also enhance the understanding of the interaction between major and minor mastitis pathogens, which may be beneficial for mastitis control programs.

## 2 Materials and methods

### 2.1 Bacteria selection and preparation

For major mastitis pathogens, one standard and two mastitis strains of each *S. uberis, S. agalactiae,* and *S. aureus* were included. The standard strains were *S. uberis* ATCC 27958, *S. agalactiae* ATCC 27956, and *S. aureus* ATCC 25923. All mastitis strains of the major pathogens were randomly selected from frozen mastitis pathogen stock (−80 °C) from the Laboratory of Milk Quality and Mastitis, the Faculty of Veterinary Medicine, Chiangmai University. Three mastitis strains of each *S. chromogenes* and *S. hominis* were used as minor mastitis pathogens and used for coculturing with the major pathogens. All mastitis strains were originally isolated from milk samples of mastitis cows. All stock of major and minor mastitis pathogens were confirmed by MALDI-TOF. The mastitis strains of minor pathogens were selected from our laboratory’s stock based on their optimal survival in coculture with *S. aureus in vitro*, as reported by Srithanasuwan et al. (2023). The selected bacteria were thawed in Tryptone Soya Broth (TSB, HIMEDIA, Mumbai, India) at 37 °C with shaking at 150 rpm for 16–18 h. Cultures were streaked onto Tryptone Soya Agar (TSA, HIMEDIA) and incubated at 37 °C for 24 h. Single colonies were suspended in 0.85% Normal Saline Solution (NSS) and adjusted to the required turbidity. The stocked major bacteria were standardized to 10^8^ colony-forming units per milliliter (CFU/mL) and subsequently diluted to 10^5^ CFU/mL. Based on the initial inoculation ratio, which influences the structure and interactions of microbial communities (Gao et al., 2020), and therefore, to benefit in the future application of the maximum protective effects of NAS against major pathogens, the stocked minor bacteria were adjusted to a maximum concentration of 10^9^ CFU/mL to enhance NAS competitiveness before being used in the experiment.

### 2.2 Milk preparation for *in vitro* culture and study design

The study was conducted separately three times for *S. uberis, S. agalactiae,* and *S. aureus*, respectively, using three major and six minor mastitis strains for each time. To minimize the effects of living organisms, such as white blood cells, other microorganisms, and specific components from immune responses, homogenized ultra-high temperature (UHT) full-fat milk was used instead of raw milk (Bai et al., 2023). Before each study, UHT commercial milk was purchased from a convenience store. For each study time, 108 sterile tubes were prepared in 27 culture conditions from 9 single cultures (3 strains of one major pathogen species and 6 strains of 2 minor pathogen species) and 18 cocultures (3 major x 6 minor), testing for 4 times at 0, 8, 12, and 24 h. For a separate culture, 4.5 mL of milk and 0.5 mL of the stock major or minor bacteria were added to the tube. For a cocultured culture, 4 mL of milk, 0.5 mL of the stock major bacteria, and 0.5 mL of the stock minor bacteria were added to the tube. In addition, 5 mL of UHT milk was used as the negative control. After well shaking, cultures were incubated at 37 °C with shaking at 150 rpm for a maximum of 24 h. The Standard Plate Count (SPC) was adapted from Anderson et al. (2011). Results were expressed as the average of counts multiplied by a dilution factor used in CFU/mL.

### 2.3 Measurement of bacterial growth in milk

After incubation for 0, 8, 12, and 24 h, each separated and cocultured milk was collected to determine the number of bacteria using the Standard Plate Count. The tubes were diluted in the range of 10^−1^ to 10^−12^ to suit each milk sample. Fifty μL of each serially diluted sample was then transferred onto sterile media using the drop plate method, with some modifications in the use of selective media according to the bacterial species. In the separation of streptococci and staphylococci, Edwards agar and modified Mannitol salt agar were used instead of plate count agar. In contrast, the separation between other staphylococci and *S. aureus* was cultured with modified mannitol salt agar and Baird-Parker agar. Colonies on Edwards agar, modified mannitol salt agar, and the grey-black, shiny, opaque zone around the colony on Baird-Parker agar were identified as streptococci, Staphylococcus spp., and *S. aureus,* respectively, and then the number of bacteria was counted. We averaged the total CFU counts of at least three drops at each counted dilution. The result was expressed as the mean of the counts multiplied by the dilution factor used in CFU/mL.

### 2.4 Statistical analysis

Data on bacterial counts were transformed into a logarithmic scale (base 10) for normality. Geometric means and their standard error of means (SE) were used for data description. The growth of major or minor mastitis pathogens in single or coculture over the culture periods of 0, 8, 12, and 24 h was analyzed using repeated measure analysis (Proc mixed, SAS university edition) due to the repeated data from one isolate. The analysis was separately conducted for each major mastitis pathogen. To normalize bacterial count data, the logarithm of bacterial counts was used as a dependent variable. Since the data from the same isolates were collected at different times, the isolate ID was defined as the repeated factor, and the correlation structure in this analysis was autoregression type 1, which was the best fit in the model. The prior statistical tests were constructed for separate dependent variables, including coculture bacteria, culture periods, and their interaction, and the interactions were significant in most models, indicating that both factors could not be separately explained. Therefore, the interaction was used as an independent variable. For the multiple comparison, least square means and SE were calculated under a fit of a heteroscedastic one-way model with Dunnett’s T3 method (Dunnett, 1980). The “pdiff” statement was used to define differences in the least-square means of bacterial counts on the log-scale among the specified times of each coculture and to demonstrate the bacterial growth patterns. To determine the effect of the overall coculture, either major or minor bacterial counts were used as a dependent variable. The culture period served as the control independent variable, and the coculture bacteria were used as the independent variable. The significant levels were defined at *p* < 0.05.

## 3 Results

**Figure 1:**
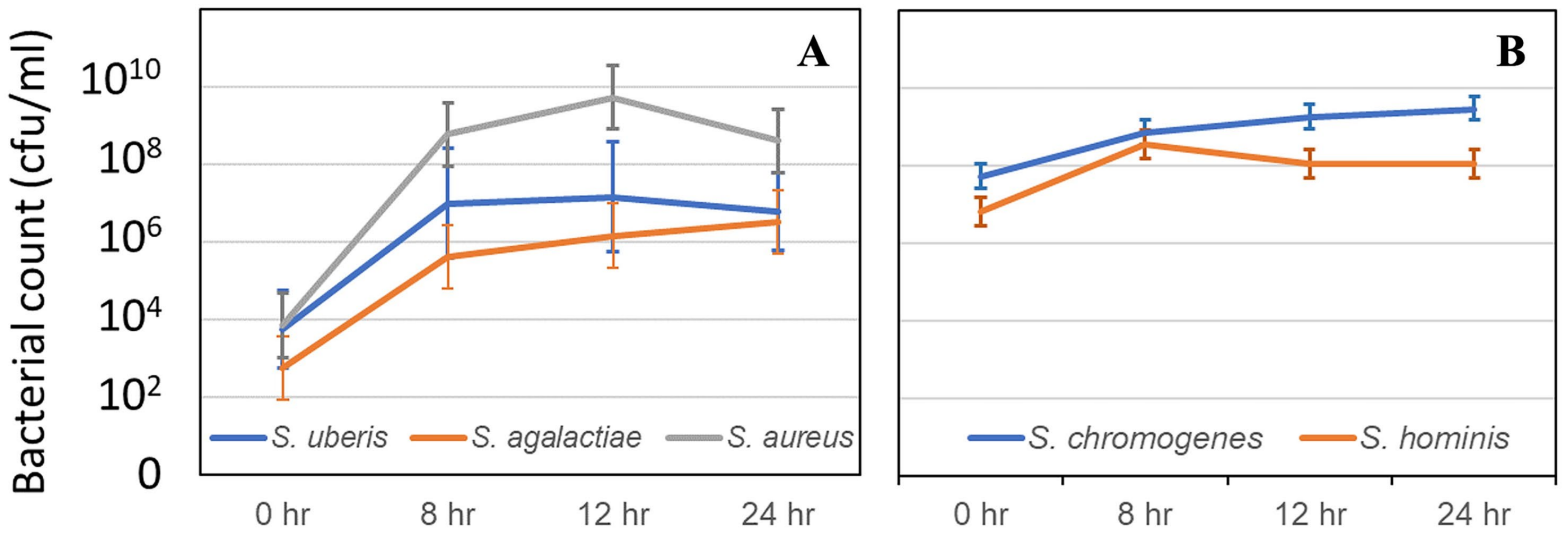
Least square means and their standard error of means (SEM) of bacterial count of mastitis pathogens (CFU/mL), including major pathogens **(A)** and minor pathogens **(B)**, after culture in milk for 0, 8, 12, and 24 h.

Averaged starting bacterial counts at 0 h for *S. uberis, S. agalactiae, S. aureus, S. chromogenes, and S. hominis* were 0.0057, 0.00095, 0.0072, 407.9, and 104.3 4 × 10^5^ CFU/mL, respectively. All major pathogens exhibited rapid growth during the first 12 h of incubation, with *S. aureus* showing the highest proliferation rate. Growth curves for each pathogen are shown in Figure 1. Patterns of bacterial counts among pathogens when cultured in milk for 24 h are shown in Figure 1. The highest bacterial counts were found for *S. aureus* at 52,553 × 10^5^ at 12 h. All major pathogens had higher rates of bacterial counts than minor pathogens when cultured in milk. Interestingly, only *S. agalactiae* and *S. chromogenes* exhibited the highest bacterial numbers at 24 h, while the others reached their highest numbers at 8 and 12 h before decreasing.

**Figure 2:**
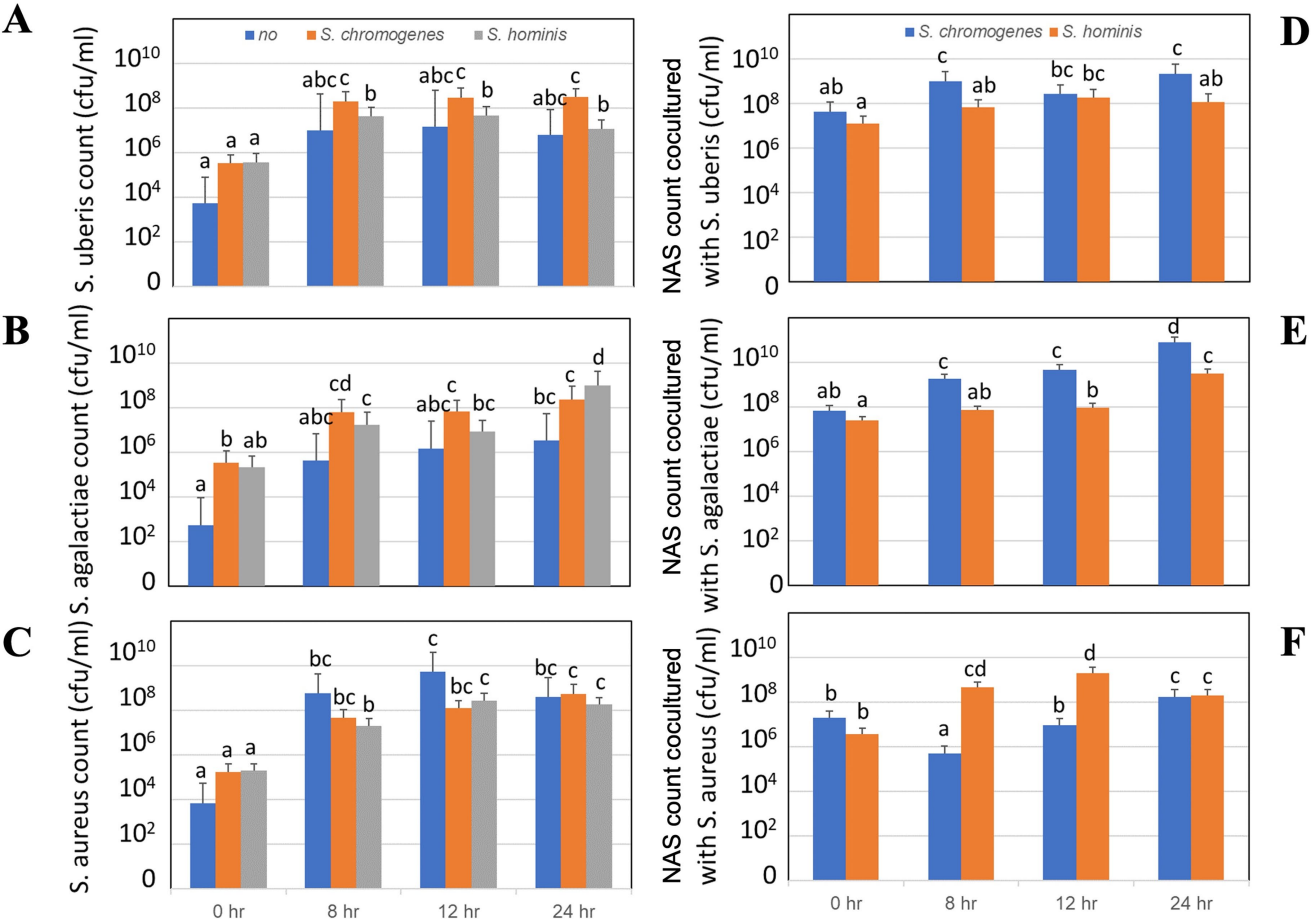
Patterns of bacterial counts of major mastitis pathogens cocultured with minor mastitis pathogens in milk for 24 h, indicated by least square means with SEM using repeated measure analysis of the mixed linear regression. **(A–C)** Growth of major pathogens: **(A)** *S. uberis*, **(B)** *S. agalactiae*, and **(C)** *S. aureus* when cocultured with *S. chromogenes* or *S. hominis*. **(D–F)** Growth of NAS: **(D)** *S. chromogenes* and **(E–F)** *S. hominis* when cocultured with major pathogens. Different letters indicate significant differences at *P* < 0.05.

The growth dynamics of *S. uberis*, *S. agalactiae*, and *S. aureus* in single and coculture with *S. chromogenes* and *S. hominis* are presented in Figure 2. For *S. uberis* (Figures 2A,D), bacterial counts increased progressively over the first 12 h in both single culture and coculture conditions, reaching approximately 10^7^–10^9^ CFU/mL. No significant suppression was observed when cocultured with NAS strains, although differences in growth rate were evident at earlier time points (8 and 12 h). Corresponding NAS counts also increased steadily in coculture, reaching up to 10^9^ CFU/mL, with *S. chromogenes* displaying slightly higher proliferation than *S. hominis* at 24 h. For *S. agalactiae* (Figures 2A,D), initial bacterial counts (~10^3^ CFU/mL) increased to 10^7^–10^9^ CFU/mL by 24 h. Coculturing with NAS did not inhibit *S. agalactiae* growth, but rather supported concurrent NAS expansion, particularly *S. chromogenes*, which reached significantly higher counts than *S. hominis* at 24 h (*p* < 0.05). For *S. aureus* (Figures 2C,F), counts increased from ~10^3^ CFU/mL at 0 h to 10^8^–10^9^ CFU/mL by 24 h. Cocultured NAS strains also proliferated robustly, with *S. hominis* exhibiting enhanced growth compared to *S. chromogenes* at 8 and 12 h, although both reached comparable levels by 24 h. Across all pathogen-NAS coculture experiments, growth of NAS strains was not suppressed by the presence of major mastitis pathogens. In several cases, NAS demonstrated a significant proliferation advantage (*p* < 0.05). Superscript letters on bars indicate statistical groupings, where different letters denote significant differences between treatments or time points.

**Figure 3:**
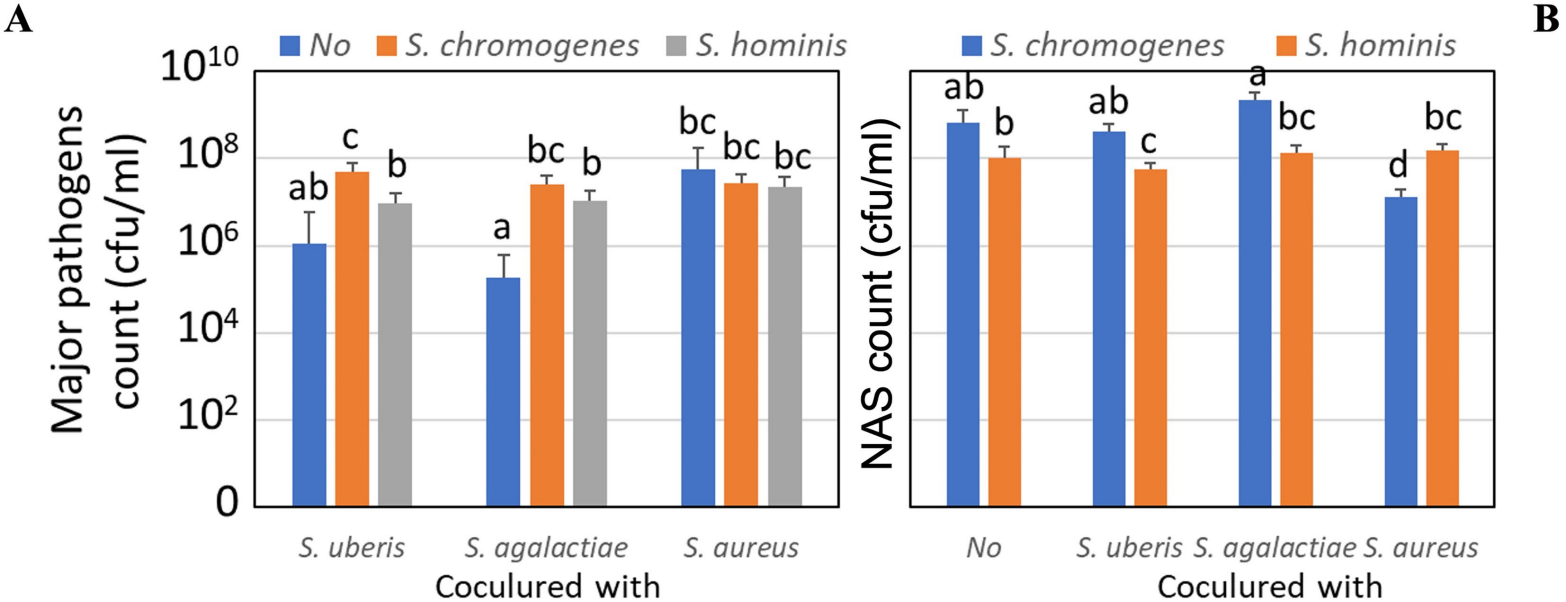
Comparisons of bacterial counts after coculturing in milk for 24 h with and without minor pathogens using least square means and their SEM calculated from the mixed linear model. **(A)** Growth of major pathogens: *S. uberis*, *S. agalactiae*, and *S. aureus* when cocultured with *S. chromogenes* or *S. hominis*. **(B)** Growth of NAS: *S. chromogenes* and *S. hominis* when cocultured with major pathogens. Different letters indicate significant differences at *P* < 0.05.

Regardless of the culture period, the coculture between major mastitis pathogens and NAS is shown in Figure 3. For major pathogen counts (Figure 3A), cocultivation with *S. chromogenes* significantly enhanced the growth of *S. uberis* compared to single culture or coculture with *S. hominis* (*p* < 0.05). In contrast, *S. agalactiae* exhibited the lowest growth when cocultured with *S. hominis*, whereas *S. chromogenes* promoted higher proliferation (*p* < 0.05). For *S. aureus*, pathogen counts were similar across treatments, with no significant suppression by the NAS strain. For NAS counts (Figure 3B), both *S. chromogenes* and *S. hominis* proliferated well in coculture with major pathogens. *S. chromogenes* reached the highest density when cocultured with *S. agalactiae*, significantly exceeding *S. hominis* (*p* < 0.05). By contrast, *S. hominis* growth was most reduced in coculture with *S. agalactiae* and *S. uberis*, although it still maintained counts in the range of 10^6^–10^7^ CFU/mL. Overall, these findings indicate species-specific interactions between NAS and major mastitis pathogens, where *S. chromogenes* generally promoted pathogen proliferation while simultaneously achieving higher own growth, particularly in the presence of *S. agalactiae.*

## 4 Discussion

The use of bacterial coculture and synthetic bacterial communities plays a crucial role in advancing our understanding of microbial ecology (Nai and Meyer, 2018), aiding in the study of species interactions, multispecies biofilms, community dynamics, and the creation of functional synthetic communities (Gao et al., 2020). When establishing a coculture system, it is crucial to select appropriate organisms and culture conditions, as well as to optimize the initial inoculation ratio. Studies in coculture systems have shown that the initial inoculation ratio influences the structure and interactions of microbial communities (Gao et al., 2020). This study utilized a significant difference in starting concentrations between NAS, set at 10^9^ CFU/mL, and major pathogens, which were at 10^5^ CFU/mL. In the clinical mastitis quarter, concentrations of *S. aureus* and NAS ranged between 10^4^ and 10^5^ CFU/mL (Juronen et al., 2018) and 10^5^–10^6^ CFU/mL (Wald et al., 2019), respectively, indicating that the rationale between major and minor pathogens was almost similar. Therefore, this discrepancy did not accurately represent the natural interspecies competition dynamics in the microbial ecology of the mastitis quarter but rather emphasized the creation of functional synthetic communities. This experimental strategy was designed to reveal potential phenomena of NAS, enhancing NAS competitiveness, which is expected to promote or inhibit major pathogens. This experimental approach, therefore, represents a novel perspective that bridges laboratory findings and the infection dynamics of different concentrations of both pathogens *in vitro*, rather than focusing on natural microbial interactions.

In this study, UHT milk was used as a culture medium to simulate bacterial growth patterns after intramammary infection of major, minor, and mixed infections. UHT milk was used to test the antimicrobial activity of the herb solution by mixing mastitis bacteria, the herb, and milk to simulate the udder environment (Suriyasathaporn et al., 2024; Negus et al., 2017). In addition, milk composition affects bacterial growth through its nutrient content, with components like fat and certain proteins potentially inhibiting growth, while others, like fatty acids, provide an energy source. Although lactoferrin, *β*-LG, and lactoperoxidase are known for their antibacterial and protective properties in milk (Piccinini et al., 2005; Hyvönen et al., 2010; Chaneton et al., 2011), high temperature of the UHT process can destroy those components in raw milk (Ludikhuyze et al., 2001). Although UHT milk provides a sterile and nutritionally rich environment for bacterial growth, it differs substantially from raw milk in several physicochemical and biological aspects. The UHT process denatures whey proteins. It also disrupts the milk fat globule membrane (MFGM). This leads to loss of bioactive components such as lactoferrin, lysozyme, immunoglobulins, and lactoperoxidase (Lee and Sherbon, 2002; Gathercole et al., 2023). These components influence bacterial adhesion, growth, and host–pathogen interactions in the mammary gland. Therefore, bacterial proliferation observed in UHT milk may not fully reflect *in vivo* infection dynamics in the udder. In the udder, immune factors and intact milk structures play key roles. Future studies using raw or minimally processed milk could provide a closer approximation to the intramammary environment.

The representative bacteria in this study were obtained from both standard strains of major pathogens and field strains of both major and minor pathogens. Differences in both phenotypic and genotypic characteristics of mastitis pathogens have been reported for *S. uberis* (Leelahapongsathon et al., 2020), *S. agalactiae* (Wataradee et al., 2023), *S. aureus*, and other staphylococci (Sophorn et al., 2025). Consistent with previous reports, *S. chromogenes* and *S. hominis* exhibit substantial genetic and phenotypic diversity at the strain level, which can influence their growth behavior and pathogenic potential under varying environmental conditions (Khasapane et al., 2025; Reydams et al., 2022). Their role in the intramammary microbial ecology is complex and multifaceted, ranging from commensalism to opportunistic pathogenicity, and even exhibiting protective effects against major mastitis pathogens. Therefore, our bacteria might be partly representative bacterial species; the results of this study should be carefully applied. Previous studies (Srithanasuwan et al., 2023; De Vliegher et al., 2004; Braem et al., 2014) mainly used artificial media to study cocultures of mastitis pathogens, which may not represent the true nutritional and physiological conditions in milk. A medium that closely mimics the intramammary environment. Future studies should include a broader range of NAS isolates to validate whether the observed patterns hold across the diversity of NAS strains in natural mastitis environments.

The Standard Plate Count (SPC) method was applied with some modifications using selective media based on the target species. Similar approaches were reported by Souto et al. (2010), indicating that counting microorganisms in bulk raw milk on Baird-Parker Agar serves as a method for detecting coagulase-positive staphylococci in bovine mastitis. Mannitol Salt Agar (MSA) was used for the differentiation between other staphylococci and *S. aureus* (Visscher et al., 2013). Meanwhile, Edwards’ crystal violet aesculin blood agar has been used for the detection of *S. agalactiae* and *S. uberis*. In general, the growth rate of specified numbers of each bacterium differed according to the medium (Morgan et al., 1941). However, the comparisons of the growth of major pathogens, *S. uberis, S. agalactiae,* and *S. aureus*, using different media, were not conducted between species, while the comparisons of *S. chromogenes* and *S. hominis* were conducted for inter-species comparisons.

This study demonstrated distinct growth dynamics of major and minor mastitis pathogens in milk (Figure 1). For the first 12 h of incubation, marked increases were observed for major pathogens, but *S. chromogenes* and *S. hominis* continued to increase slightly. These different growth patterns in milk environments were supported by earlier reports on the differential growth kinetics of mastitis pathogens (Bradley, 2002; Rainard and Foucras, 2018). In this study, major pathogens exhibited higher bacterial growth rates compared to minor pathogens, which might be related to their pathogenic potential and ability to adapt to milk as a nutrient source (Keane, 2019). The rapid proliferation of *S. uberis*, *S. agalactiae,* and *S. aureus* within 12 h as the regular milking interval might be related to persistent infection and partly clinical mastitis, for example, persistent *S. uberis* and *S. agalactiae* (Leelahapongsathon et al., 2016; Leelahapongsathon et al., 2020). In addition, the aggressive growth behavior of *S. aureus* during the early hours might contribute to its frequent association with acute clinical mastitis (Zecconi and Scali, 2013). In contrast, the slow proliferation of *S. chromogenes* and *S. hominis* might cause subclinical transient mastitis for most minor pathogens (Srithanasuwan et al., 2024).

Our findings show that the bacterial growth was varied among major mastitis pathogens when cocultured with the minor pathogens (Figures 2A–C, 3A). The growth rate of *S. uberis* was higher when cocultured with *S. chromogenes* than in its single culture; however, the growth rate of *S. agalactiae* was higher when cocultured with both minor pathogens (Figure 3A). The antagonistic activity of *S. uberis* against *S. chromogenes* involves the production of bacteriocins, such as nisin U, a variant of the broad-spectrum bacteriocin nisin (Wirawan et al., 2006), and ubericin A (Heng et al., 2007), which has proven activity against various Gram-positive bacteria, including staphylococci. No significant difference was found for the growth rate of *S. aureus* after coculture. In support of our *in vitro* study using standard media culture, we found that major pathogens were able to destroy most NAS (Srithanasuwan et al., 2023), indicating that the results might vary among bacterial strains. In a study, only two out of 10 *S. chromogenes* isolates from two different teats from the same cow consistently reduced the growth of all *S. aureus*, *S. uberis,* and *S. dysgalactiae* strains (De Vliegher et al., 2004). *S. chromogenes* plays a substantial role in promoting the growth of major mastitis pathogens, potentially via mechanisms such as metabolite release or altered nutrient availability in milk. In contrast, in an *in vivo* murine study, prior experimental intramammary inoculation of *S. chromogenes* reduced subsequent *S. uberis* inoculation growth and increased cytokine levels compared to a single *S. chromogenes* IMI (Vander Elst et al., 2023). In the specific case of bovine mastitis, recent insights suggest the use of bovine NAS to prime the mammary gland and prevent infection by major mastitis pathogens (Matthews et al., 1990; De Vliegher et al., 2004; Braem et al., 2014). This reduction in major mastitis pathogens may be due to the increase in immune response following the mixed infection (Chaisri et al., 2024; Vander Elst et al., 2023).

Except for the higher growth of *S. hominis* cocultured with *S. aureus*, the bacterial growth of *S. chromogenes* in both single and coculture with most major pathogens was higher than that of *S. hominis* (Figures 2D–F, 3B). This result is consistent with the work of Braem et al. (2014), who reported the strongest inhibitory effect of *S. chromogenes* against a wide range of mastitis-causing pathogens and others. Additionally, several mechanisms of *S. chromogenes* inhibit the growth of major pathogens (Chin et al., 2021; Carson et al., 2017; Rahmdel et al., 2019). Bacteriocin production is a strain- not species-specific trait (Nakatsuji et al., 2017). However, selecting *S. hominis* for the highest survival when cocultured with *S. aureus* might lead to increased growth of *S. hominis* in milk. In support of our previous study, *in vitro* culture of *S. hominis* at 24 h prior to coculture with *S. aureus* could survive, but not for *S. chromogenes* (Srithanasuwan et al., 2023). The use of high concentrations of *S. chromogenes* and *S. hominis* at about 10^8^ CFU/mL compared to 10^4^ of *S. aureus* might imitate the situation of prior culture of NAS, allowing the growth of *S. hominis* before the coculture.

This study found that major mastitis pathogens, such as *S. uberis*, *S. agalactiae*, and *S. aureus*, interacted in different ways as found in the conventional culture media with the proven NAS protective strains, like *S. chromogenes* and *S. hominis*. These findings suggest new strategies for testing an innovation for mastitis pathogens within a milk-based environment before using experimental animals.

## 5 Conclusion

In conclusion, the in vitro high concentration of the proven protective NAS strains could survive in the milk environment after coculture with major pathogens, with some modifications, as no reduction effect was observed. This study also showed that major mastitis pathogens proliferated in milk culture more quickly than minor pathogens, which were tested with much higher concentrations. We also conclude that the experimental ecological interactions between major and minor mastitis pathogens in milk depend on their species, incubation times, and strains. For example, *S. chromogenes* significantly boosts the growth of both *S. uberis* and *S. agalactiae*, while *S. hominis* only increases the proliferation of *S. agalactiae*. Therefore, this milk culturing method offers a practical in vitro approach to studying interactions between mastitis pathogens. It could also serve as a tool for future studies evaluating coculture interactions in milk, expanding to various bacteria and strains to identify beneficial microorganisms for health. However, the significantly different concentrations used for both major and minor pathogens could not explain natural bacterial interactions. To evaluate the natural bacterial interaction, future research with the proper rationale concentrations of bacteria needs to be explored.

## Data Availability

The original contributions presented in the study are included in the article/supplementary material, further inquiries can be directed to the corresponding author.
